# Biweekly cetuximab in combination with FOLFOX-4 in the first-line treatment of wild-type *KRAS* metastatic colorectal cancer: final results of a phase II, open-label, clinical trial (OPTIMIX-ACROSS Study)

**DOI:** 10.1186/1471-2407-14-865

**Published:** 2014-11-22

**Authors:** Julen Fernandez-Plana, Carlos Pericay, Guillermo Quintero, Vicente Alonso, Antonieta Salud, Miguel Mendez, Mercedes Salgado, Eugeni Saigi, Luis Cirera, on behalf of the ACROSS Study Group

**Affiliations:** Hospital Universitario Mútua Terrassa, Terrassa, Spain; Corporación Sanitaria Parc Taulí, Sabadell, Spain; Hospital Universitario Lucus Augusti (HULA), Lugo, Spain; Hospital Miguel Servet, Zaragoza, Spain; Hospital Arnau de Vilanova, Lleida, Spain; Hospital de Móstoles, Móstoles, Spain; Complejo Hospitalario Universitario de Ourense, Ourense, Spain; Corporación Sanitaria Parc Taulí, Sabadell, Spain; Hospital Universitario Mútua Terrassa, Plaça del Dr. Robert N°5, Terrassa, Barcelona 08221 Spain

**Keywords:** Cetuximab, FOLFOX-4, Metastatic colorectal cancer, First-line, Wild-type *KRAS*

## Abstract

**Background:**

This phase II study aims to evaluate the efficacy and safety of biweekly cetuximab in combination with oxaliplatin, leucovorin, and fluorouracil (FOLFOX-4) as first-line treatment of metastatic wild-type *KRAS* colorectal cancer.

**Methods:**

Previously untreated patients with wild-type *KRAS* tumours received biweekly cetuximab (500 mg/m^2^ on day 1) plus FOLFOX-4 (oxaliplatin 85 mg/m^2^ on day 1, leucovorin 200 mg/m^2^ on days 1 and 2, and fluorouracil as a 400 mg/m^2^ bolus followed by a 22-hour 600 mg/m^2^ infusion on day 1 and 2). Treatment was continued until disease progression, onset of unacceptable toxicities, metastases surgery, or discontinuation request. The primary endpoint was ORR.

**Results:**

The intention-to-treat population included 99 patients with a median age of 64.1 years (range, 34-82). The ORR was 60.6% (95% CI, 50.3% to 70.3%). The median follow-up was 17.8 months; the median OS and PFS were 20.8 and 10.1 months, respectively. Metastases from colorectal cancer were surgically resected in 26 (26.3%) patients, with complete resection achieved in 18 (69.2%) patients. Median PFS and OS in patients undergoing metastatic resection were 12.6 and 29.5 months, respectively. The most common grade 3-4 toxicities were neutropenia (32.3%), acne-like rash (15.2%) and diarrhoea (11.1%).

**Conclusions:**

The efficacy of the biweekly combination of cetuximab with FOLFOX-4 in patients with wild-type *KRAS* tumours supports the administration of cetuximab in a dosing regimen more convenient for patients and healthcare providers. The activity of the biweekly administration is similar to what has been reported for the weekly regimen. Reported toxicity was also consistent with the known toxicity profile of weekly cetuximab.

**Trial registration:**

EudraCT Number 200800690916

## Background

Colorectal cancer (CRC) is the second most common cancer and the second leading cause of cancer mortality in Europe [1]. The major cause of death in CRC are distant metastases [2]. It is expected that approximately 25% of patients diagnosed with CRC present with metastasis at initial diagnosis, whereas approximately 50% of patients will develop metastatic CRC (mCRC) during the follow-up [3].

Significant advances in the treatment of mCRC have been made within the last years after decades of only modest progress with 5-fluorouracil (5-FU) monotherapy. Consequently, the combination of oxaliplatin or irinotecan with 5-FU have markedly improved treatment outcomes [4–6]. Furthermore, the addition of targeted therapies to conventional mCRC chemotherapy regimens has resulted in further improvement of efficacy results [7].

Cetuximab, a chimeric monoclonal antibody that targets the epidermal growth factor receptor (EGFR), is currently a component of the standard of care for mCRC [8]. Two randomised clinical trials demonstrated the clinical efficacy of adding weekly cetuximab to irinotecan- or oxaliplatin-based chemotherapy regimens in the first-line treatment of patients with wild-type *KRAS* mCRC [9–12].

The standard cetuximab dosing regimen, both as a monotherapy and in combination with chemotherapy, involves an initial intravenous infusion of 400 mg/m^2^ with subsequent weekly doses of 250 mg/m^2^. In contrast, a biweekly dosing schedule -every 14 days- would offer several advantages in terms of convenience and a more economical use of healthcare resources [13]. Moreover, these benefits would be enhanced in mCRC treatment regimens as standard first-line chemotherapy regimens approved for use in combination with cetuximab in wild-type *KRAS* mCRC, such as oxaliplatin, 5-FU infusion and leucovorin (FOLFOX) or irinotecan plus 5-FU infusion and leucovorin (FOLFIRI), are already administered in a biweekly basis.

The feasibility of a biweekly cetuximab administration schedule was demonstrated in a two-part phase I dose-escalation study [14]. This study demonstrated that cetuximab can be safely administered as single agent or in combination with FOLFIRI at doses between 400 and 700 mg/m^2^ in a biweekly schedule, and 500 mg/m^2^ was established as the recommended dose on the basis of pharmacokinetic exposure data [14]. Furthermore, data provided by several studies involving a combined regimen of cetuximab and irinotecan support the hypothesis that safety and efficacy of a biweekly schedule are similar to a weekly schedule [15–17].

Seeking to increase convenience for patients and healthcare providers, this phase II study was designed with the aim to evaluate the efficacy and safety of biweekly cetuximab in combination with FOLFOX-4 in the first-line treatment of wild-type *KRAS* mCRC.

## Methods

### Study design

This multicentre, single-arm, open-label, phase II clinical trial was carried out in 15 Spanish centres (EudraCT Number: 2008-006909-16). The local authorities and ethic committees or institutional review boards at each participating centre approved the study protocol and its amendments. The study was conducted in accordance with the ethical principles of the Declaration of Helsinki. All patients provided written informed consent.

### Patients

Inclusion criteria were an age of 18 years of older, histologically confirmed colorectal carcinoma, wild-type *KRAS* tumours, first occurrence of metastatic disease, at least one radiologically measurable lesion, a life expectancy of ≥12 weeks, an Eastern Cooperative Oncology Group (ECOG) Performance Status ≤1, and adequate hematologic, hepatic and renal function. Patients with prior exposure to anti-EGFR therapy or chemotherapy for metastatic disease (with the exception of oxaliplatin if completed ≥6 months prior to inclusion) were not eligible for inclusion.

### Study treatment

Patients received a biweekly intravenous (IV) infusion of cetuximab (500 mg/m^2^ on day 1) followed by FOLFOX-4 (2-hour oxaliplatin 85 mg/m^2^ infusion on day 1 in tandem with a 2-hour leucovorin 200 mg/m^2^ infusion on day 1 and 2, and 5-FU as a 400 mg/m^2^ bolus followed by a 22-hour 600 mg/m^2^ infusion on day 1 and 2). Cetuximab was administered over 2 hours in the first cycle, over 1.5 hours in the second cycle and over 1 hour thereafter. Appropriate prophylactic medication was administered to prevent the occurrence of acute hypersensitivity reactions before each cetuximab administration.

Protocol dose modifications were permitted in the event of predefined toxic effects related to chemotherapy or cetuximab [17]. In the event of unacceptable toxicity due to 5-FU/leucovorin, oxaliplatin, or cetuximab, the agent responsible could be discontinued and the patient could continue with the other study medications. However, protocol modifications did not allow the maintenance of oxaliplatin as a monotherapy or in combination with cetuximab. Treatment was continued until disease progression, onset of unacceptable toxic effects, a patient/physician request to discontinue, or surgery for metastases.

### Study assessments

Pre-treatment evaluations included the determination of *KRAS* mutation status. Mutation analysis was performed centrally at the Hospital Universitario Mútua Terrassa. Tumour DNA was extracted from formaldehyde-fixed paraffin-embedded tissues. Mutant *KRAS* in exon 2 was detected using a validated *KRAS* mutation kit (DxS LTD., Manchester UK) that identifies seven somatic mutations located in codons 12 and 13 using allele-specific real-time PCR. The analysis was performed in an ABI Prism 7300 instrument (Applied Biosystems).

Computed tomography (CT) or magnetic resonance imaging (MRI) was performed at baseline, every 8 weeks during the first 6 months of the study, and every 12 weeks thereafter until disease progression. Adverse events were collected throughout the study period. All adverse events recorded were graded according to the Common Toxicity Criteria of the National Cancer Institute (CTC-NCI) version 3.0.

### Statistical analysis

The primary endpoint of the study was the objective response rate (ORR) defined according to the Modified Response Criteria in Solid Tumours (RECIST 1.1) [18]. Secondary endpoints included progression-free survival (PFS), duration of response, overall survival (OS) and toxicity profile of biweekly cetuximab in combination with FOLFOX-4. Patients who underwent surgery for metastases were censored at the date of surgery in the PFS analysis. Median PFS and OS following metastasectomy were also assessed for this group of patients. The cut-off date for collection of survival data was November 19^th^, 2012.

A sample size of 98 patients was calculated to detect a 95% confidence interval (CI) for the ORR of 50-70%, assuming an estimated rate of 60% according to previous studies and an anticipated 10% of patient loss to follow-up [9].

The intention to treat population (ITT) included all patients that received at least one dose of the combination chemotherapy (four drugs) and had at least one radiological assessment at 8 weeks. Safety analysis was conducted in the group of patients that received at least one dose of any of the four drugs (oxaliplatin, leucovorin, 5-FU or cetuximab).

Numerical variables were summarized as mean and standard deviation (SD). For categorical variables, absolute and relatives frequencies were calculated. Time-to-event variables were analysed using the Kaplan-Meier method. Relative dose intensity (RDI) was calculated by dividing the dose intensity of the administered regimen by the dose intensity of the drug in the standard planned regimen. Data analysis was performed using the Statistical Analysis System version 9.2 (SAS 9.2).

## Results

### Patient disposition and baseline characteristics

From July 2009 until December 2011, 101 patients were included in the study. Two patients immediately withdrew their consent. The ITT population consisted of 99 patients. Safety analysis was carried out on the 99 patients who received at least one dose of any component of the study treatment. Demographic and clinical characteristics at baseline are shown in Table 1. Median age of the patients was 64.1 years (range: 34-82 years). Thirty-five patients presented with metastases limited to the liver. The median duration of follow-up was 17.8 months.

**Table 1:** **Demographic and clinical data at baseline in the intention-to-treat population**

Characteristic	N = 99
Male, n (%)	66 (66.7)
White/Caucasian ethnicity, n (%)	98 (99.0)
Median age, years (range)	64.1 (34-82))
ECOG status, n (%)	
0	51 (51.5)
1	48 (48.5)
Tumor status at diagnosis, n (%)	
T1	1 (1.0)
T2	6 (6.1)
T3	43 (43.4)
T4	25 (25.3)
Unknown	24 (24.2)
Node status at diagnosis, n (%)	
N0	22 (22.2)
N1	24 (24.2)
N2	25 (25.3)
Unknown	28 (28.3)
Metastases status at diagnosis, n (%)	
M0	21 (21.2)
M1	76 (76.8)
Unknown	2 (2.0)
Primary tumor site, n (%)	
Colon	59 (59.6)
Rectum	40 (40.4)
Metastases sites*, n (%)	
Liver	87 (87.9)
Lung	39 (39.4)
Lymph nodes	27 (27.3)
Other	17 (17.1)
Prior therapy, n (%)	
Adjuvant chemotherapy (FOLFOX)	9 (9.1%)
Neoadjuvant chemo-radiotherapy for rectal cancer	7 (7.1%)
Surgery	48 (48.5%)

*Abbreviations*: *ECOG* Eastern Cooperative Oncology Group, *ITT* Intention to treat, *Q1-Q3* Interquarile range.

*A patient may had metastases in more than one organ.

### Efficacy

The best confirmed ORR was 60.6% (95% CI, 50.3% to 70.3%) (4 complete and 56 partial responses) (Table 2). Thirty patients (30.3%) had stable disease. Therefore, the disease control rate (DCR) was 90.9% (95% CI, 83.4% to 95.8%). In the 60 patients with a partial or complete response, median time to onset of response was 1.9 months and median duration of response was 8.6 months.

**Table 2:** **Efficacy in the intention-to-treat population**

Variable	ITT population N = 99
Response, n (%)	
Complete response	4 (4.0)
Partial response	56 (56.6)
Stable disease	30 (30.3)
Progressive disease	5 (5.1)
Not available	4 (4.0)
ORR, % (95% CI)	60.6 (50.3 to 70.3)
DCR, % (95% CI)	90.9 (83.4 to 95.8)
Progression-free survival	
Progression event, n (%)	68 (68.7)
Months of progression-free survival, median (Q1-Q3)	10.1 (6.5-14.8)
Overall survival	
Death, n (%)	57 (57.6)
Months of overall survival, median (Q1-Q3)	20.8 (11.6-32.8)

*Abbreviations*: *CI* confidence interval, *DCR* disease control rate, *ITT* intention-to-treat, *ORR* objective response rate, *Q1-Q3* Interquarile range.

**Figure 1:**
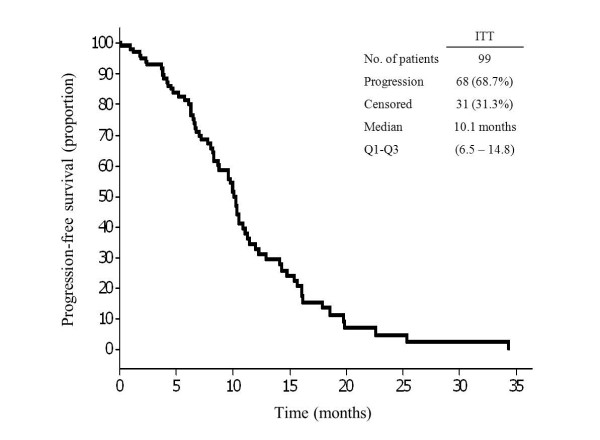
**Kaplan-Meier plot for progression-free survival in the intention to treat (ITT) population.**

**Figure 2:**
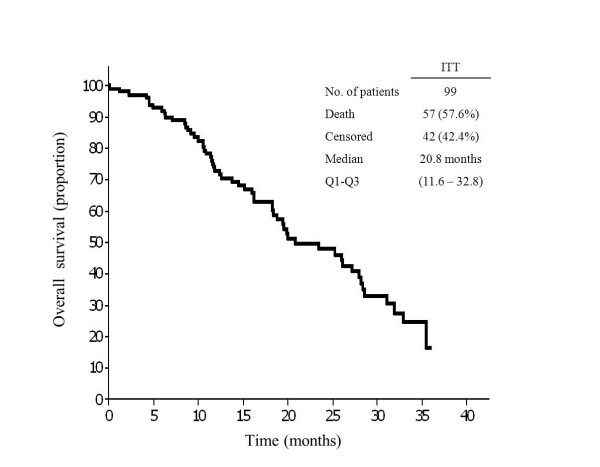
**Kaplan-Meier plot for overall survival in the intention to treat (ITT) population.**

Objective disease progression was observed in 68 patients. Median PFS was 10.1 months (Q1-Q3, 6.5-14.8) (Table 2; Figure 1). Time to progression, defined as time from the first treatment administration to the first objective disease progression in the 60 patients who had a complete/partial response, was 10.4 months (Q1-Q3, 6.8-17.9). At the time of data collection cut-off there were 57 deaths (57.6%). The median OS was 20.8 months (Q1-Q3, 11.6-32.8) (Table 2; Figure 2).

Twenty-six patients (26.3%) underwent surgery for liver metastases, with complete surgical resection (R0) observed in 18 (18.2%) patients. The median PFS and OS after surgical resection of metastases were 12.6 months and 29.5 months, respectively. Twenty-one patients (80.8%) who underwent surgery for metastases received adjuvant chemotherapy. Thirteen of the 21 patients continued with cetuximab-containing regimens after surgery.

### Treatment exposure

The median duration of cetuximab treatment was 20.9 weeks (range: 1-148). The median cumulative dose of cetuximab was 4659.4 mg/m^2^ and the median intensity of dose per cycle was 447.1 mg/m^2^; therefore the median cetuximab dose used in this study was similar to the recommended 500 mg/m^2^ dose. Consequently, the median relative dose intensity was 89%, with a total of eighty-two patients (82.8%) receiving ≥80% relative dose intensity (RDI) of cetuximab. Thirty-nine patients (39.4%) had at least one cetuximab dose reduction. Delays in cetuximab dosing were mostly due to skin reactions. As for chemotherapy, the median duration of oxaliplatin and 5-FU treatment was 18.3 weeks (range: 1-46) and 22.9 weeks (range: 1-83), respectively. A RDI ≥80% was achieved in 58 patients (58.6%) for oxaliplatin and in 55 patients (55.6%) for 5-FU. Two or more lines of treatment were received by 62.6% of patients, whereas 5.1% of patients underwent surgery and did not receive additional lines of treatment post-surgery.

### Safety

Adverse events (AEs) of any grade occurring in at least 10% of patients and grade 3 or 4 AEs occurring in at least 2% of patients are summarized in Table 3. Seventy-seven patients (77.8%) presented at least one grade 3 or 4 AE. The most common grade 3 or 4 AEs were neutropenia (32.3%) and diarrhoea (13.1%).

**Table 3:** **Adverse events**

Adverse event	Any grade	Grade 3 or 4
Any adverse event*	97 (98.0%)	77 (77.8%)
**Blood and lymphatic system disorders**		
Anaemia	11 (11.1%)	2 (2.0%)
Febrile neutropenia	6 (6.1%)	5 (5.1%)
Leukopenia	15 (15.2%)	1 (1.0%)
Neutropenia	49 (49.5%)	32 (32.3%)
Thrombocytopenia	23 (23.2%)	2 (2.0%)
**Gastrointestinal disorders**		
Abdominal pain	30 (30.3%)	2 (2.0%)
Diarrhoea	58 (58.6%)	13 (13.1%)
Intestinal obstruction	8 (8.1%)	8 (8.1%)
Nausea	28 (28.3%)	2 (2.0%)
Stomatitis	52 (52.5%)	5 (5.1%)
Vomiting	21 (21.2%)	1 (1.0%)
**General disorders and administration site disorders**		
Asthenia	65 (65.7%)	8 (8.1%)
**Immune system disorders**		
Drug hypersensitivity	9 (9.1%)	3 (3.0%)
**Metabolism and nutrition disorders**	37 (37.4%)	4 (4.0%)
Anorexia	25 (25.3%)	2 (2.0%)
**Musculoskeletal and connective tissue disorders**		
Back pain	8 (8.1%)	2 (2.0%)
**Nervous system disorders**		
Dysaesthesia	11 (11.1%)	2 (2.0%)
Dysgeusia	12 (12.1%)	1 (1.0%)
Peripheral neuropathy	11 (11.1%)	2 (2.0%)
Neurotoxicity	22 (22.2%)	2 (2.0%)
Paraesthesia	40 (40.4%)	6 (6.1%)
**Psychiatric disorders**		
Anxiety	3 (3.0%)	2 (2.0%)
**Respiratory, thoracic and mediastinal disorders**		
Pulmonary embolism	3 (3.0%)	2 (2.0%)
**Skin and subcutaneous tissue disorders**		
Dry skin	23 (23.2%)	4 (4.0%)
**Vascular disorders**		
Deep vein thrombosis	3 (3.0%)	2 (2.0%)
**Special adverse event categories**		
Acne-like Rash	91 (91.9%)	15 (15.2%)
Infusion related reaction^a^	14 (14.1%)	0 (0.0%)
Nail toxicity^b^	28 (28.3%)	6 (6.1%)

*Listed are adverse events of any grade occurring in at least 10% of patients and adverse events of grade 3 or 4 occurring in at least 2% of patient.

^a^This special event category included the preferred terms infusion related reaction and pyrexia.

^b^This special event category included the preferred terms nail toxicity and paronychia.

For grade 3 or 4 special adverse events, 15.2% patients presented with acne-like rash, 6.1% presented with nail toxicity (this special event category included the preferred terms nail toxicity and paronychia) and no patient presented with infusion-related reactions (this special event category included the preferred terms “infusion related reaction” and “pyrexia”). Hypomagnesemia was reported in only one patient (grade 4). Cetuximab was discontinued in 33 patients (33.3%), oxaliplatin was discontinued in 41 patients (41.4%) and 5-FU was discontinued in 25 patients (25.3%) due to adverse events.

Disease progression was the primary cause of death (91.2% of deaths). Two patients died due to an unrelated AE and one patient died due to a related AE. This patient presented with pulmonary fibrosis that was considered related to oxaliplatin and/or cetuximab.

## Discussion

This study showed that the efficacy of biweekly-administered cetuximab in combination with FOLFOX-4 is similar to what has been reported for the standard weekly cetuximab dosing regimen as first-line treatment of wild-type *KRAS* mCRC. The ORR obtained in the present study (61%) was similar to the figure reported in a previous phase II study with a weekly administration of cetuximab in combination with FOLFOX-4 [10]. A similar ORR was also obtained (62%) in the biweekly cetuximab arm of the recently published randomised study performed by the Central European Co-operative Oncology Group (CECOG) [19]. In the weekly cetuximab arm of that phase II study, a lower ORR was obtained (53%). The CECOG trial was not powered to establish non-inferiority of biweekly administration versus weekly administration of cetuximab, so further studies would be needed to confirm their findings.

The median PFS (10.1 months) observed is also in line with the 8.3 months and 9.5 months reported in previous studies with weekly cetuximab combined with FOLFOX-4, and very similar to the figure obtained in the biweekly arm of the CECOG trial (9.2 months) [10, 19]. It was also slightly longer than the range of 8.4-9.1 months reported for standard weekly cetuximab in combination with other oxaliplatin-based chemotherapy regimens in patients with wild-type *KRAS* mCRC [7, 20]. Although the short median follow-up of our study does not permit us to draw definitive conclusions regarding OS, the median time obtained (20.8 months) was slightly shorter but consistent with the median OS reported for standard weekly cetuximab in combination with FOLFOX-4 and in the biweekly arm of the CECOG trial [10, 19].

Complete surgical resection of colorectal liver metastases is potentially curative and provides clear survival benefits in patients with disease confined to the liver. Within this context, 35 out of 99 patients in our study presented with only liver metastases. 26.3% of patients underwent surgery for colorectal liver metastases and a R0 resection rate was achieved in 18% of patients. This R0 resection rate is higher than that reported in the cetuximab weekly arm (5%) and in the cetuximab every second week arm (10%) of the CECOG trial [10, 19]. However, in contrast to the CECOG trial metastases resectability was not a selection criterion in the present study. Therefore, while some patients were initially candidates for surgery, other patients presented with unresectable metastases. In our study, surgical resected patients presented a median OS considerably longer than unresected patients. These results confirm the improved prognosis of patients undergoing surgery for colorectal liver metastases.

The toxicity profile of the biweekly administration of cetuximab is also consistent with the known safety profile of the standard weekly cetuximab dosing regimen. The incidence rate of grade 3 or 4 AEs was in line with that reported previously for cetuximab in combination with FOLFOX-4 [10, 18, 19]. Therefore, a higher dose of cetuximab administered on an every-2-weeks basis is not associated with a greater incidence of grade 3 or 4 adverse events. As expected, the most common grade 3 or 4 adverse events were neutropenia and acne-like rash. The high proportion of patients that required at least one cetuximab dose reduction are attributable to dose adjustments planned in case of dose delay of chemotherapy due to haematological toxicity.

## Conclusions

In conclusion, despite the known limitations of a non-randomised single arm study, our results support the administration of cetuximab in a dosing regimen more convenient for both patients and healthcare providers. The option to synchronise the administration of cetuximab and chemotherapy would reduce the number of administration visits. Such reductions would improve patient’s quality of life and simplify treatment administration for healthcare workers. Additionally, the reduction in the number of visits would reduce the costs of treatment of patients with mCRC without reducing the efficacy previously observed for cetuximab combined with FOLFOX-4.

## Abbreviations

5-FU: 5-fluorouracil
AE: Adverse Event
CEGOG: Central european cooperative oncology group
CI: Confidence interval
CRC: Colorectal cancer
CT: Computed tomography
CTC-NCI: Common toxicity criteria of the national cancer institute
DCR: Disease control rate
ECOG PS: Eastern cooperative oncology group performance status
EGFR: Epidermal growth factor receptor
FOLFIRI: Irinotecan plus infusional 5-fluorouracil and leucovorin
FOLFOX: Oxaliplatin plus infusional 5-fluorouracil and leucovorin
ITT: Intention to treat population
IV: Intravenous
mCRC: Metastatic colorectal cancer
MRI: Magnetic resonance imaging
ORR: Objective response rate
OS: Overall survival
RDI: Relative dose intensity
RECIST: Response criteria in solid tumours
SD: Standard deviation.
